# Cell spheroid fusion: beyond liquid drops model

**DOI:** 10.1038/s41598-020-69540-8

**Published:** 2020-07-28

**Authors:** Nastasia V. Kosheleva, Yuri M. Efremov, Boris S. Shavkuta, Irina M. Zurina, Deying Zhang, Yuanyuan Zhang, Nikita V. Minaev, Anastasiya A. Gorkun, Shicheng Wei, Anastasia I. Shpichka, Irina N. Saburina, Peter S. Timashev

**Affiliations:** 1 FSBSI “Institute of General Pathology and Pathophysiology”, 8, Baltiyskaya st., Moscow, 125315 Russia; 2 FSBEI FPE “Russian Medical Academy of Continuous Professional Education” of the Ministry of Healthcare of Russia, 2/1, Barrikadnaya St., Moscow, 125993 Russia; 3 Faculty of Biology, Lomonosov Moscow State University, 12-1, Leninskie Gory, Moscow, 119234 Russia; 4 Institute for Regenerative Medicine, Sechenov First Moscow State Medical University, 8-2, Trubetskaya St., Moscow, 119991 Russia; 5 Institute of Photonic Technologies, Research Center “Crystallography and Photonics” RAS, 2, Pionerskaya st., Troitsk, Moscow, 142190 Russia; 6 Department of Urology, Children’s Hospital of Chongqing Medical University, Chongqing, People’s Republic of China; 7 Wake Forest University Institute for Regenerative Medicine, Winston-Salem, NC USA; 8 Department of Oral and Maxillofacial Surgery/Central Laboratory, Peking University School and Hospital of Stomatology, Beijing, 100081 China; 9 Laboratory of Biomaterials and Regenerative Medicine, Academy for Advanced Interdisciplinary Studies, Peking University, Beijing, 100871 China; 10 Department of Polymers and Composites, N.N. Semenov Institute of Chemical Physics, 4, Kosygin st., Moscow, 119991 Russia; 11 Chemistry Department, Lomonosov Moscow State University, 1‑3, Leninskiye Gory, Moscow, 119991 Russia

**Keywords:** Regenerative medicine, Mesenchymal stem cells, Stem-cell research

## Abstract

Biological self-assembly is crucial in the processes of development, tissue regeneration, and maturation of bioprinted tissue-engineered constructions. The cell aggregates—spheroids—have become widely used model objects in the study of this phenomenon. Existing approaches describe the fusion of cell aggregates by analogy with the coalescence of liquid droplets and ignore the complex structural properties of spheroids. Here, we analyzed the fusion process in connection with structure and mechanical properties of the spheroids from human somatic cells of different phenotypes: mesenchymal stem cells from the limbal eye stroma and epithelial cells from retinal pigment epithelium. A nanoindentation protocol was applied for the mechanical measurements. We found a discrepancy with the liquid drop fusion model: the fusion was faster for spheroids from epithelial cells with lower apparent surface tension than for mesenchymal spheroids with higher surface tension. This discrepancy might be caused by biophysical processes such as extracellular matrix remodeling in the case of mesenchymal spheroids and different modes of cell migration. The obtained results will contribute to the development of more realistic models for spheroid fusion that would further provide a helpful tool for constructing cell aggregates with required properties both for fundamental studies and tissue reparation.

## Introduction

Modern approaches to the rapidly evolving fields of regenerative medicine and tissue engineering are closely associated with the development and formation of tissue-engineered constructions, where cellular components play a crucial role^1–3^. Monolayer cell culture is the most widely used approach to the growing and studying of cells in vitro. Nevertheless, 2D culture conditions cause cell flattening and remodeling of the cell’s internal structure, which can eventually affect the gene expression^4^. On the other hand, 3D cell culture better reflects the in vivo microenvironment both morphologically and physiologically. The extra dimension which 3D cell cultures have, compared to monolayers, helps to establish intercellular junctions, to reorganize the cytoskeleton, to polarize and to differentiate in conditions similar to native tissue conditions^5^. Multicellular spheroids obtained under non-adhesive conditions represent one possible 3D cell culture system. There is a great deal of unexplored potential in spheroid-based research, as tissue engineering using spheroids is a relatively new field^6–8^.

Three-dimensional bioprinting of scaffold-based and scaffold-free tissue-engineered constructions is widely used for tissue substitution and modeling of organs-on-chips^9–12^. Cell spheroids with prefabricated intercellular junctions and extracellular matrix provide a new promising type of bioinks suitable for processing by an automated biofabrication technology^8^. Predictive modeling of fusion of spheroids from different cell types is necessary to optimize the printing parameters and thus enhance the quality of the final products.

Blindness and visual impairment, common targets for regenerative medicine, are mostly associated with macular diseases, such as age-related macular degeneration affecting the outer retina. Glaucoma, which affects the inner retina, and diabetic retinopathy, which ultimately affects all retinal layers, are among other highly prevalent retinal diseases. Cell-based therapies in ophthalmology are based on the combined use of epithelial cells [such as retinal pigment epithelium (RPE) cells] and mesenchymal stem cells (MSCs)^13^. The RPE is believed to play a central role in retinal diseases associated with aging^14^. Modeling RPE functional changes, for example basolateral functions, that take place in pathological states like in cases of macular degeneration are more preferable in the form of 3D culture and cell spheroids^15^. The limbal stroma of the eye is one of the promising sources of MSCs^16^. Bioprinting approaches in ophthalmology are used primarily in reconstruction of the anterior segment with the production of artificial cornea and lens that are unique and patient-specific in shape and design^17^. The further development of the technology of bioprinting of individual components of the eye as well as the whole functional organ is impossible without the modernization of bio-inks. We suppose that cellular spheroids from epithelial and mesenchymal stem cells of eye tissues can become such bio-ink. In addition to obtaining/bioprinting tissue-engineered constructions, further maturation with the rapid correct formation of functional tissue is also essential^18^. As long as scaffold-free spheroids have prefabricated intercellular junctions and extracellular matrix, which promotes the preservation of cell viability and developmental plasticity, using them, not single-cell suspension, as building blocks in bioprinting will stimulate the formation and maturation of the obtained tissue-engineered constructions^19,20^.

Understanding how cells form tissues and organs^21^ is one of the significant challenges of tissue engineering and developmental biology. The fusion of cell aggregates has become a widely used technique for studying this matter^22^. Past experiments have explored the analogy of cell aggregate fusion with the fusion of liquid droplets^23–26^, which might be seen as a part of the larger concept of tissue liquidity^27–29^. According to this concept, the main parameters that determine the fusion process are the viscosity and surface tension^30^. Cell aggregates, however, are much more complicated than liquid drops, which will affect the limits of the liquid drop model applicability. Indeed, while the biomechanical properties of the cytoskeleton mostly determine the mechanical properties of individual cells in isolation, the material properties of multicellular spheroids and tissues arise through complex associations of cell adhesion molecules with each other, the cytoskeleton and the extracellular matrix. The mechanical measurements on single spheroids are associated with technical difficulties due to small size of the spheroids and conditions required for the maintenance of its viability^31–33^. Moreover, most studies in the field of material properties of multicellular spheroids have focused only on immortalized cell lines^32,34,35^, and there has been no detailed investigation of spheroids from primary cultures of human somatic cells.

In the present research, we studied the fusion process of spheroids from two primary human somatic cell cultures of mesenchymal (limbal mesenchymal stem cells—L-MSCs) and epithelial (retinal pigment epithelium cells—RPE cells) phenotypes. We also conducted a thorough analysis of the morphology and mechanical properties of the spheroids. In contrast to the predictions of the liquid drop model, the fusion was faster in the spheroids from epithelial cells with lower surface tension, indicating that the simple liquid drop analogy does not always work. We proposed hypotheses about why the model fails in this case; supposedly due to the unaccounted-for role of the ECM.

## Results

### Characterization of the Monolayer and 3D Cultures of L-MSCs and RPE Cells

**Figure 1:**
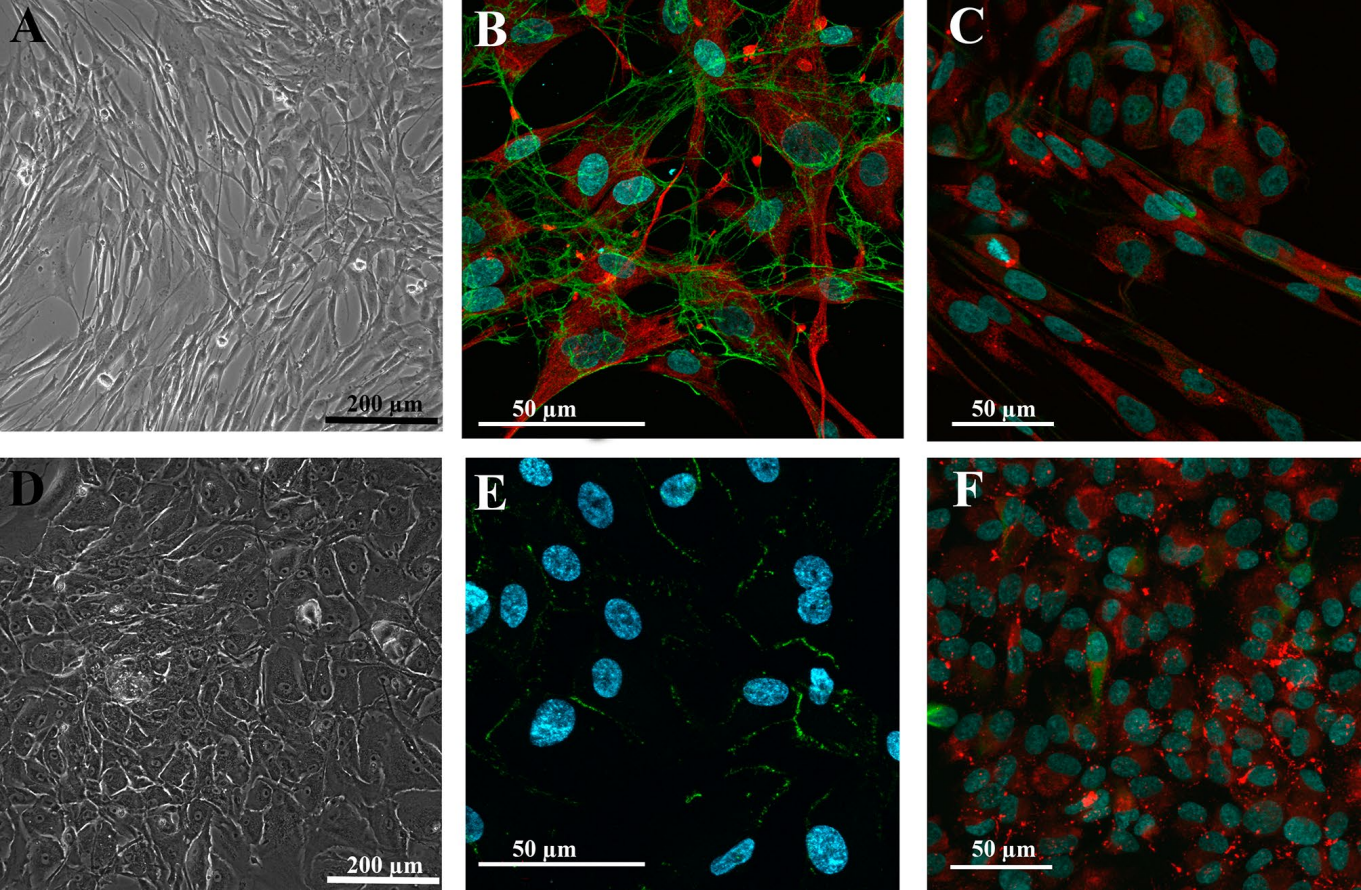
The morphology of the monolayer cell culture of the L-MSCs (**A**–**C**) and the RPE cells (**D**–**F**). (**A**)—the mesenchymal morphology of the L-MSC culture at the fourth passage; (**B**)—expression of the mesenchymal marker vimentin (red) and the extracellular matrix component fibronectin (green); (**C**)—laminin (red) expressed only in the cytoplasm. In a few cells low expression of nestin (green) was observed; (**D**)—RPE cells at the fourth passage formed a cobblestone-like monolayer, which is characteristic of epithelial cells; (**E**)—RPE cells showed membrane distribution of the epithelial tight junction marker ZO-1 (green); (**F**)—the component of basal membranes laminin (red) was expressed in cytoplasm and between cells. The expression of nestin (green) was present in only a few cells. (**A**, **D**)—*light phase-contrast microscopy*; (**B**, **C**, **E**, **F**)—*laser scanning confocal microscopy*, nuclei (blue) stained with Hoechst 33,258.

In the 2D culture, L-MSCs had a typical spindle-like shape (Fig. 1A) and formed a monolayer in three to five days, while keeping a high proliferative potential till the fourth passage, expressing mesenchymal markers (CD105, CD90 and CD29) and very low level of haematopoietic and lymphocytic markers (Table 1, Supplementary Fig. S1). Expression of the mesenchymal marker vimentin and synthesis of the extracellular matrix component fibronectin were also found in MSC monolayers (Fig. 1B), while the epithelial basal membrane marker laminin was expressed only in cytoplasm, and the marker of undifferentiated cells, nestin, was present just in a few cells (Fig. 1C).

**Table 1:** Expression of surface markers in 2D (fourth passage) and 3D (seven days) L-MSC and RPE cell cultures.

	L-MSCs		RPE cells	
	2D culture*	7-day spheroids**	2D culture*	7-day spheroids**
CD11b	8.2%	25.7%	6.3%	20.8%
CD14	0.8%	3.3%	6.2%	0.7%
CD19	5.0%	15.7%	40.0%	1.5%
CD29	99.9%	95.3%	99.9%	100.0%
CD34	5.3%	4.4%	0.5%	11.4%
CD90	99.8%	43.9%	83.1%	98.5%
CD45	2.4%	3.2%	43.5%	27.6%
CD105	86.2%	94.3%	58.9%	74.0%

*The value of each sample consisted of 1 × 10^6^ cells.

**The value of each sample consisted of 750 spheroids.

RPE cells showed a cobblestone appearance, characteristic of epithelial cells in a 2D culture (Fig. 1D). The monolayer formed in five to seven days and the proliferative potential was preserved until the fourth passage. The pigment melanin was present in the primary culture, but its expression decreased during passaging. In confluent RPE cell cultures, tight junctions formed between the cells, which was confirmed with immunostaining for ZO-1 (Fig. 1E). Laminin was expressed both in the cytoplasm and between the cells, though only a few cells showed nestin expression (Fig. 1F). At the fourth passage, 83.1% of the RPE cells were CD90-positive, and 58.9% were CD105-positive (Table 1, Supplementary Fig. S2). The relatively high level of expression of the MSC markers CD29, CD90 and CD105 in RPE 2D cultures indicates that cells undergo an epithelial-mesenchymal transition in monolayer culture and acquire an intermediate epithelial-mesenchymal phenotype.

**Figure 2:**
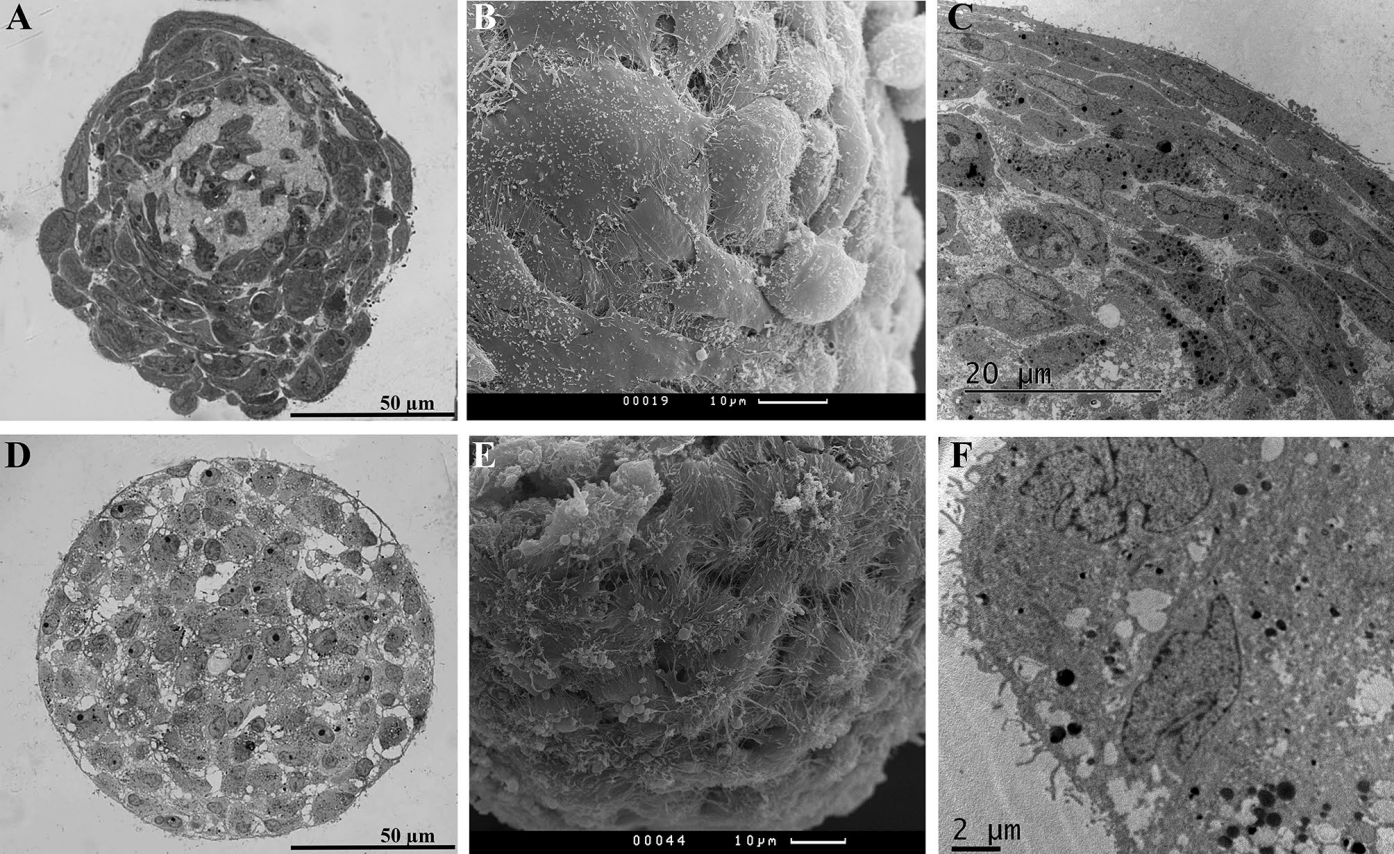
Structure of seven-day spheroids from the L-MSCs (**A**–**C**) and RPE cells (**D**–**F**). (**A**)—the dense outer zone of L-MSC spheroids was formed from several layers of imbricated flattened cells. In the inner area, cells were round or polygonal, embedded in an extracellular matrix; (**B**)—surface of the L-MSC spheroids; (**C**)—elongated cells of the outer zone and a part of the inner zone of L-MSC spheroids; (**D**)—the outer zone of RPE-cell spheroids was formed with cells that partially restored apical-basal polarity. Cells in the inner area were round or polygonal, located loosely with a low amount of extracellular matrix; (**E**)—the surface of the RPE-cell spheroids; (**F**)—surface cells of RPE spheroids with microvilli. (**A**, **D**)—s*emi-fine sections, light microscopy*; (**B**, **E**)—*scanning electron microscopy*; (**C**, **F**)—*transmission electron microscopy.*

**Figure 3:**
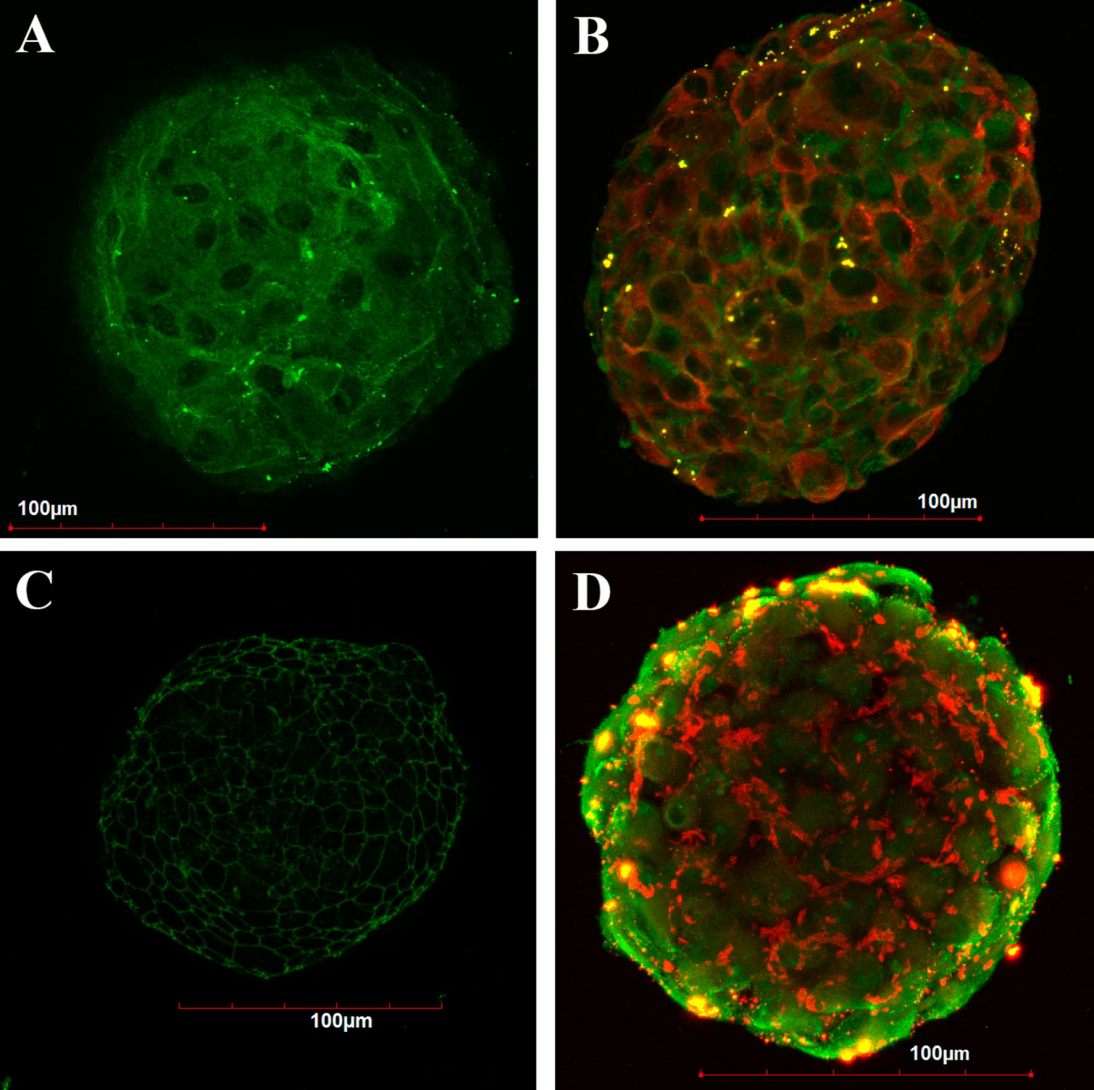
Expression of markers in seven-day spheroids from the L-MSCs (**A**, **B**) and RPE cells (**C**, **D**). (**A**)—vimentin; (**B**)—laminin in the cytoplasm (red) and nestin (green) in few cells. (**C**)—tight junctions with an expression of ZO-1; (D)—laminin in the cytoplasm and between cells (red) and nestin (green) in the cells of a surface layer. *Laser scanning confocal microscopy.*

Under non-adhesive conditions, cells tend to form multicellular structures—spheroids that differ in their morphology depending on the initial cell type. Spheroids from cells of the mesenchymal phenotype consisted of several surface layers of elongated, densely packed cells and an inner zone with polygonal cells and an extracellular matrix (Fig. 2A–C, Supplementary Fig. S3A, B). Cells in L-MSC spheroids had a well-developed cytoskeleton represented by intermediate filaments of vimentin (Fig. 3A), and they synthesized the extracellular matrix component laminin, which was found both in the cytoplasm and in the extracellular space (Fig. 3B, red). More cells expressed nestin, compared to the 2D culture (Fig. 3B, green).

In contrast, the surface zone of RPE-cell spheroids consisted of one or two layers of epithelial-like cells with intercellular junctions and partially restored apical-basal polarity. Cells in the inner area were round, and the extracellular matrix was less abundant (Fig. 2D–F, Supplementary Fig. S3C). The epithelial phenotype of surface cells in seven-day spheroids from RPE cells was confirmed by immunostaining for the tight junction protein ZO-1 (Fig. 3C). Surface cells, as opposed to inner cells, were also characterized by expression of the stem cell marker nestin (Fig. 3D, green). Laminin was present both in the cytoplasm and between cells but was less abundant than in L-MSC spheroids (Fig. 3D, red).

### Mechanical properties of the monolayers and Spheroids from L-MSCs and RPE Cells

L-MSC and RPE-cell monolayer cultures had very similar values of Young’s modulus, as measured by nanoindentation (Table 2, Supplementary Fig. S4). While the L-MSC spheroids had a similar stiffness to that of the L-MSC monolayer, the RPE-cell spheroids were ~ 2.3 times softer than the RPE-cell monolayers. The observed high values of the standard deviation can be related to the biological properties of the studied objects and indentation of different cell compartments, as well as the regions of intercellular junctions. The viscoelastic analysis revealed the similar behavior of spheroids and monolayers (Table 2). The power-law rheology (PLR) model was selected here for the description of the spheroid properties. The relaxation function for the power-law rheology (PLR) model was selected in this study as:

1 $$ E(t) = E_{1} t^{- \alpha },$$

**Table 2:** The values of Young’s modulus (kPa) and the power-law exponent in 2D and 3D cultures; mean ± standard deviation.

	2D monolayer	7-day-old spheroid
L-MSCs		
Young’s modulus	3.92 ± 0.63	3.94 ± 0.81
Power law exponent	0.15 ± 0.03	0.17 ± 0.04
RPE cells		
Young’s modulus	3.82 ± 0.82	1.63 ± 0.34*
Power law exponent	0.15 ± 0.04	0.16 ± 0.03

*p < 0.001 versus other groups.

where *E*_*1*_ is the scale factor of the relaxation modulus (relaxation modulus at *t* = 1 s) and *α* is the power-law exponent. A larger *α* value means a larger amount of relaxation; materials exhibit a solid-like behavior at *α* = 0 and a fluid-like behavior at *α* = 1. The *E*_*1*_ value characterizes the sample stiffness in a manner similar to the Young’s modulus, but less dependent on the indentation speed. A close fit with the model was obtained for the complete set of indentation curves (Supplementary Fig. S11). As opposite to the viscoelastic function with several relaxation times^36^, the PLR and other fractional calculus models allow to characterize relaxation of biological materials with less number of independent parameters^37^.

The values of the power-law exponent, a parameter which characterizes the amount of relaxation, were similar in all the samples, with a slight tendency to increase in seven-day spheroids.

The similar stiffness of L-MSC monolayers and spheroids indicates the presence of active tensional homeostasis^38^. Presumably, the cells can modify the surrounding environment, via ECM and other cells in external layers of the spheroid, until its properties are equal to their characteristic stiffness phenotype^39,40^. The same level of tension in L-MSCs on hard plastic and in the spheroid is further confirmed by the similar values of the power-law exponent *α*, which characterizes the viscoelastic behavior. Since RPE-cell spheroids are 2.4 times softer than spheroids from L-MSCs, we might expect a lower tension in the surface layer. The tension *σ* is proportional to the Young’s modulus *E*, and might be estimated as *Γ* ~ *E·R*_*0*_, where *R*_*0*_ is the spheroid radius^41,42^. Thus, approximately three times higher tension is expected in the L-MSC spheroids. This is in agreement with a previous study, where higher surface tension was found in spheroids composed of human skin fibroblasts (mesenchymal phenotype) then in spheroids composed of epithelial CHO (Chinese hamster ovary) cells^43^. On the other hand, the absence of a large quantity of extracellular matrix in RPE-cell spheroids might make them overall softer than L-MSC spheroids.

The differences in mechanical properties between RPE-cell spheroids and monolayers may originate from the differences in cell phenotypes. The cells on the surface of the spheroid revert to the epithelial phenotype, while some fraction of the cells on the culture plastic had the mesenchymal phenotype, which is known to be stiffer than the epithelial^44,45^. In the monolayer 2D culture, RPE cells lose their hexagonal shape and pigment granules and become polygonal, and the integrity of intercellular junctions is compromised. Liggett et al. have described this phenomenon during the obtaining of the bovine RPE immortalized cell line^46^. It has also been shown, using atomic force microscopy (AFM) on porcine RPE-cell monolayer cultures, that cell stiffness depends on the presence of melanosomes containing melanin. The Young’s modulus of non-pigmented cells was 4.98 ± 0.17 kPa, which was three times lower than in pigmented cell cultures^47^. Our values of Young’s modulus, obtained for the RPE-cell monolayer culture at the fourth passage, when cells are almost non-pigmented and are polygonal or elongated, are consistent with data described in these previous studies and are close to the values obtained for L-MSC monolayer cultures.

### Study of cell spheroid fusion

**Figure 4:**
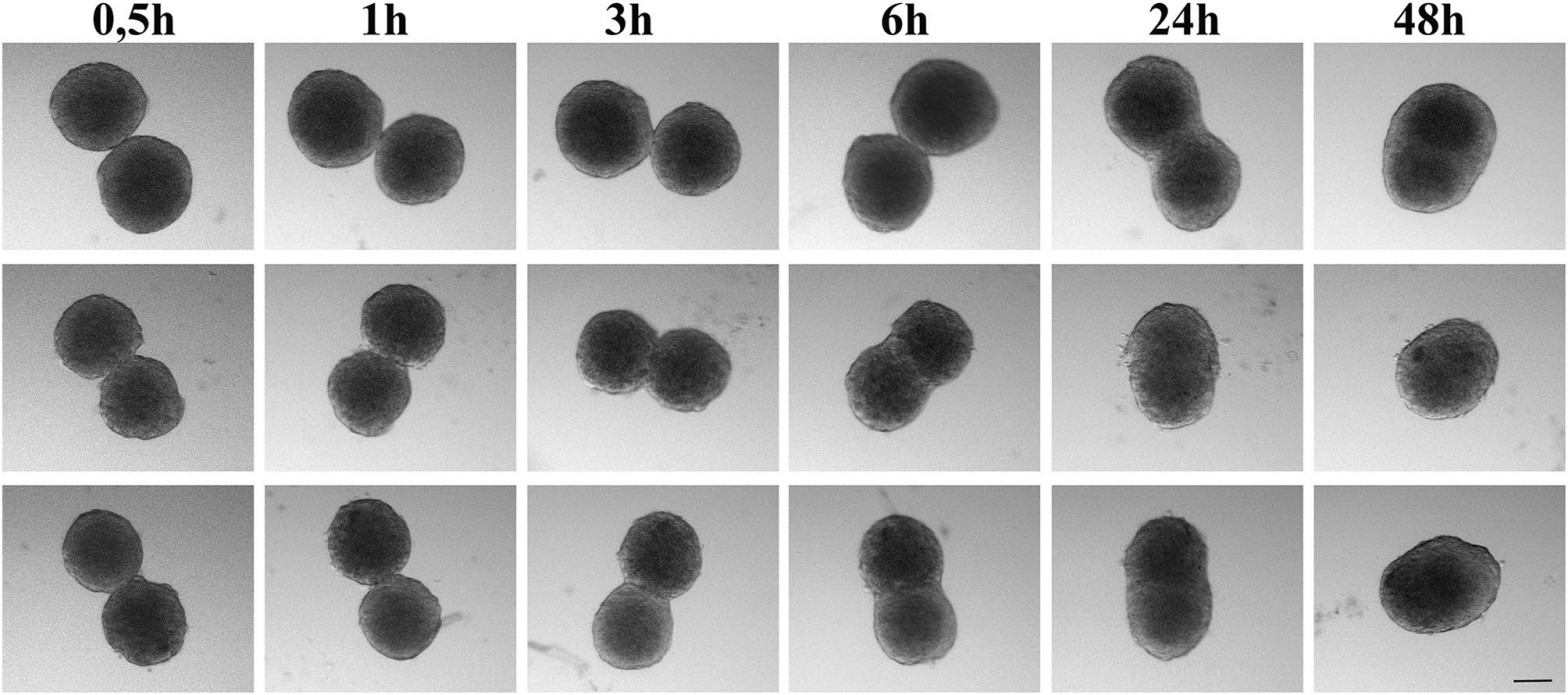
The dynamics of spheroid fusion within 48 h in a hanging drop system. Two L-MSC spheroids (upper row), two RPE-cell spheroids (middle row) and L-MSCs with RPE-cell spheroids (lower row). Scale bar: 100 µm.

**Figure 5:**
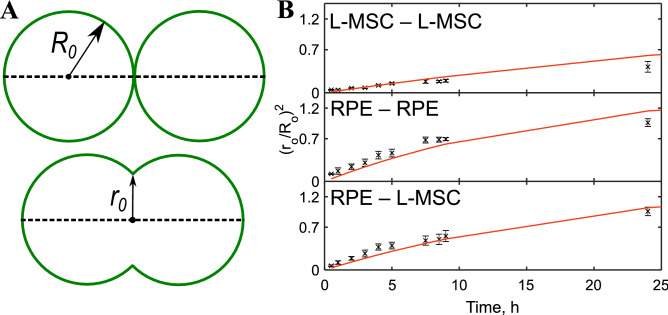
Fusion of the 7-day-old spheroids analyzed with the applied model for liquid drops. (**A**)—schematic drawing showing the measured parameters, the initial average radii *R*_*0*_ of the spheroids in pairs and the neck radius *r*_*0*_ during spheroid fusion. (**B**)—time evolution of the parameter (*r*_*0*_/*R*_*0*_*)*^2^ during the fusion of two L-MSCs cells (top), two RPE (middle) and an RPE–L-MSC pair of spheroids; an average value of 5 different spheroid pairs with similar initial radii (vertical bars represent SDs). Fitting of the data sets with the model is shown with red curves (The Adj. R^2^ values are 0.91, 0.65, 0.91 for L-MSC–L-MSC, RPE–RPE and RPE–L-MSC, respectively).

The fusion process was noticeably faster for the 7-day-old RPE-cell spheroids than for L-MSC-cell spheroids. From the time-lapse observation (Fig. 4), the neck formation and the fused area extension went faster in the former case. Fusion of the spheroids was quantitatively analyzed using the model of the coalescence of highly viscous liquid drops under the action of surface tension^26^, which has been widely used in previous studies^23,24,48,49^. The model predicts that the squared normalized neck radius (Fig. 5A) evolves with time *t* according to the exponential function:

2 $$ \left( {\frac{{r_{0} }}{{R_{0} }}} \right)^{2} = 2^{\frac{2}{3}} \left( {1 - e^{{ - \frac{t}{\tau }}} } \right), $$

where *r*_*0*_ is the neck radius, *R*_*0*_ is the initial average radii of spheroids in pairs and *τ* is the associated time constant. From the earlier theoretical results for the coalescence of highly viscous liquid drops, the time constant is:

3 $$ \tau = 2^{\frac{2}{3}} R_{0} \frac{\eta }{\Gamma }, $$

where* η* is the viscosity of the spheroid and *Γ* is the effective surface tension. The ratio *Γ*/*η* is referred to as the visco-capillary velocity. The liquid drop model (Eq. 2) can fit the fusion data sets reasonably well for all spheroid pairs with Adj. R^2^ (adjusted coefficient of determination) values 0.91, 0.65, 0.91 for L-MSC–L-MSC, RPE–RPE and RPE–L-MSC, respectively (Fig. 5B). Moreover, time constants τ, obtained from the fits were proportional to the initial average radii *R*_*0*_ of the spheroids in the fusion pair in agreement with the Eq. (3) (*R*_*0*_ was in a range 30–120 µm). However, we have not found a significant correlation between the visco-capillary velocity and the *R*_*0*_ for all spheroid pairs (*p* > 0.15). Thus, the *Γ*/*η* ratio was used as a measure of the fusion kinetics and was found to be 3.8 ± 1.4, 8.4 ± 2.1 and 7.7 ± 2.7 µm/h for L-MSC–L-MSC, RPE–RPE and RPE–L-MSC fusion, respectively (12 fusion pairs were analyzed for each case), indicating a faster fusion of RPE-cell spheroids. The fusion speed for the heterotypic pair of spheroids (RPE–L-MSC) was almost as high as for RPE–RPE pair (Fig. 4, Supplementary Movie). From the mechanical measurements, however, we would expect a higher fusion speed in L-MSC spheroids with higher surface tension, suggesting that some other mechanisms could play a major role in the fusion process.

Additionally, we analyzed the fusion of the 3-day-old spheroids. Due to the ongoing compactization (size reduction) of the spheroids, the liquid drop model does not describe the fusion quite effectively: the neck radius is shrinking over time together with the size of the spheroids. The beginning of the fusion (first 6 h), however, follows the model quite well (Fig. S5). Using only thirst several time points of the fusion kinetics (up to 6 h), we found the values of *Γ*/*η* ratio to be 10.6 ± 4.5, 10.8 ± 1.0 and 10.5 ± 1.5 µm/h for L-MSC–L-MSC, RPE–RPE and RPE–L-MSC fusion, respectively (5 fusion pairs were analyzed for each case). Thus, all spheroid types fused with a similar speed, and for L-MSC–L-MSC pair it was markedly higher than for the same 7-day-old spheroids. The data are presented in Fig. S5 and Table 3. The apparent viscosity *η* was calculated from the *Γ*/*η* ratio based on the surface tension assessed from indentation experiments. High values of the apparent viscosity for L-MSC–L-MSC pair and its further increase for 7-day-old spheroids might indicate slow matrix remodelling processes during the fusion.

**Table 3:** The parameters of the spheroid fusion obtained with the model for liquid drops.

Pair	Initial diameter, µm	Time constant *τ*, h	Visco-capillary velocity *Γ*/*η*, µm/h	Apparent viscosity, *η* Pa*h
L-MSC–L-MSC				
3-day-old	250 ± 10	12 ± 5	10.6 ± 4.5	~ 50,000
7-day-old	250 ± 100	34 ± 16	3.8 ± 1.4	~ 200,000
RPE–RPE				
3-day-old	160 ± 10	7 ± 1	10.8 ± 1.0	~ 15,000
7-day-old	200 ± 100	12 ± 6	8.4 ± 2.1	~ 25,000
L-MSC–RPE				
3-day-old	230 ± 10	9 ± 2	10.5 ± 1.5	–
7-day-old	250 ± 100	16 ± 9	7.7 ± 2.7	–

## Discussion

Our data indicate that the liquid droplet fusion model could not be used successfully to compare the fusion of two types of spheroids from epithelial and mesenchymal cells. According to the model, the mesenchymal cells, which are able to generate much higher tension and are known to have higher migratory capabilities, should complete the fusion of spheroids much faster than the epithelial cells. Indeed, it was shown previously that apparent surface tension is about thrice higher for spheroids composed of human skin fibroblasts then of epithelial CHO cells; and faster fusion was observed between the former ones^43^. However, the opposite tendency was observed here experimentally, for the first time, to the best of our knowledge. The mechanisms of spheroid fusion might differ depending on cell origin and phenotype. Therefore, further studies involving more cell types will expand our knowledge about tissue formation processes and will allow optimizing bioink properties.

**Figure 6:**
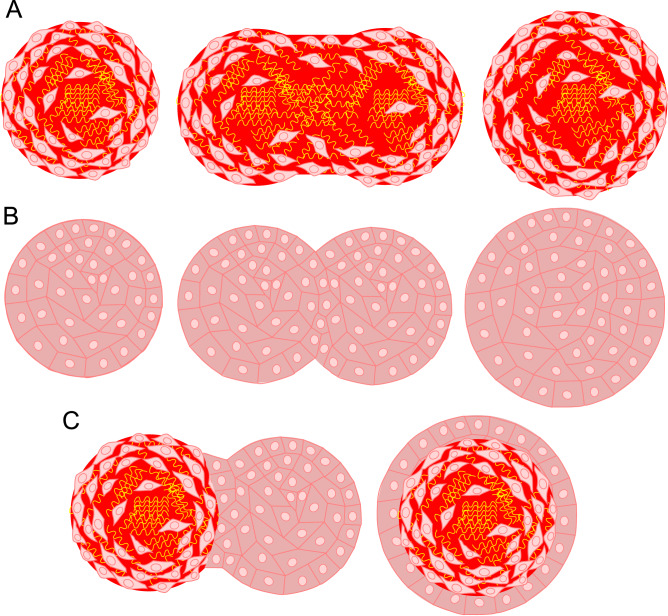
The proposed different mechanisms of fusion of spheroids from mesenchymal and epithelial cells. (**A**)—L-MSC spheroids have a large quantity of ECM, and the spheroid fusion is slow due to the required ECM remodeling; (**B**)—RPE-cell spheroids show faster fusion where collective cell rearrangements are not hampered by the ECM presence; (**C**)—fusion of L-MSC with RPE-cell spheroids proceeds fast through migration of cells from RPE spheroid on the surface of dense L-MSC spheroid without ECM remodeling. *ECM—extracellular matrix.*

The features of the fusion of L-MSC and RPE-cell spheroids observed in our study, we believe, may originate from the ultrastructure of these spheroids, specifically due to the amount of ECM and cell packaging. A large amount of ECM and dense cell packaging in the outer layer make the fusion of L-MSC spheroids slower, while loose cell packaging and a low amount of ECM result in faster fusion of RPE-cell spheroids (Fig. 6, Supplementary Fig. S6). L-MSCs have to remodel the matrix at the later stages of fusion, to recover the spherical shape, as can be seen from the intermediate elongated fusion products. We also observed much faster fusion of younger 3-day-old L-MSC spheroids that have a smaller amount of the synthesized ECM. In a recent study^50^, where the spheroids from primary sheep chondrocytes were studied, a slower fusion kinetic was observed in the older spheroids with a more significant accumulation of ECM as well. It is also possible that collective migration is occurring more efficiently in the epithelial cells with well-developed intercellular junctions^51^. The specific type of cell migration might also cause some deviation from the liquid drop model, which was observed here as a lower fit quality for the pair of RPE spheroids (Adj. R^2^ = 0.65 versus 0.91 for the other pairs).

Most of the current cell self-assembly models, including some very advanced ones^11,52^, completely ignore such biophysical factors as remodeling of the ECM and different cell migration modes in this process. Then the tissue liquidity concept is applied to the cell aggregates, the surface tension term is used with assumptions of weak cell–cell interactions, high motility and low amount of ECM. The failure of any of these conditions lead to an elastoplastic-like behaviour for the aggregate where the plastic part might be due to ECM remodelling or rearrangement of cell–cell junctions. Therefore, in presence of large amount of ECM the surface tension hypothesis is no longer valid and the models like liquid drop fusion will not give adequate predictions. An elasto-visco-plastic models of cell aggregates are now developed for the compression of the cell aggregates^53,54^, but they have not been applied yet for the fusion experiments. The ECM is expected be important in the plastic reorganizations during compression.

The ECM is known to play an essential role in tumor formation, wound healing and organ development. When properly accounted for, we believe it will provide a better understanding of these phenomena, which might be especially important for bioprinting using the MSC-cell spheroids. This, however, is not a simple task and require knowledge of such parameters as the amount of ECM in spheroid, characteristic deposition, degradation, and remodeling times and some other related parameters.

Epithelial-mesenchymal interactions, as well as tissue fusion, are the processes involved in both development and regeneration. The existing in vitro organoid models consider either formation of spheroids from the mixed suspension of different types of cells^55^ or the addition of epithelial cell suspension to the pre-formed mesenchymal spheroids, which results in the formation of the mesenchymal core covered with epithelial layers^56^. Epithelial cells do not migrate inside the mesenchymal spheroids because the existing structure composed of cells and extracellular matrix serves as a basal membrane and supporting stroma. Fusion of such structures led to the removal of epithelial cells from the seams between organoids so that at the end of the fusion epithelial cells were restricted to the outside of the resulting tissue while the mesenchymal compartment was still inside the structure^56^. We assume that in our case the fast fusion of heterotypic L-MSC and RPE-cell spheroids may go through the resembling mechanisms with epithelial cells migrating over the dense mesenchymal cell layers (Supplementary Movie, Supplementary Fig. S6B, C) without remodeling its existing structure. In this case, the resulting structure would also be in accordance with the differential adhesion hypothesis, which claims that tissue with higher surface tension will be surrounded by the one with the lower surface tension^22^. The surface tension can be defined by the number of intercellular junctions^57^ or by the Young’s modulus, as in our study. The revelation of more distinct mechanisms of cell migration in different types of spheroid fusion as well as the structure of the mixed fusion products requires further studies. As long as spheroids from single cell type have proven themselves to be a useful tool for studying tissue morphogenesis in vitro^58^ and for obtaining tissue-engineered constructions with high cell density^6,59^, the further studies in the field of fusion of spheroids from different cell types will provide a new data on how to optimize the procedures of obtaining in vitro more complex tissue-engineered constructions that would more precisely reproduce the native tissue structure and function.

Studying the mechanical properties of spheroids is a new field of research. Previous studies of material properties of cell spheroids were conducted using methods such as AFM, cavitation rheology, MicroSquisher, and microtweezers. The study of the mechanical properties of single spheroids is problematic, due to the small size of the objects—150–200 µm in diameter, with a spherical shape, which hampers their fixation on a surface, as well as the presence of a water cushion between the object and the substrate. AFM analysis was used to determine Young’s modulus of spheroids from the 3T3 murine fibroblast cell line. In each experimental group, five spheroids were measured, and after averaging the data from five-fold measurements of every spheroid, Young’s modulus was estimated to be in the range 0.3–3.5 kPa^60^. In another study, Young’s modulus of the human colon adenocarcinoma (epithelial) cancer cell line LS174T, measured by AFM, was found to be in the range 0.3–0.6 kPa, depending on the indentation speed^35^. The cavitation rheology method was used to analyze the mechanical properties of spheroids from the transformed cell line HEK 293 that continuously grow in 3D cultures due to cell proliferation. In this case, the critical pressure necessary to break the connection between cells in spheroids was estimated to be in the range 0.013–0.5 kPa^34^. Mechanical analysis of tumor spheroids has been conducted with microtweezers, and the Young’s moduli of spheroids grown from three breast cell lines (two malignant and one non-malignant) were 0.2 ± 0.06, 0.4 ± 0.09 and 1.25 ± 0.32 kPa, respectively^32^. To date, there is only one study that has investigated the mechanical properties of spheroids from human bone marrow-derived mesenchymal stem cells using a CellScale MicroSquisher. The average Young’s modulus of two-day mesenspheres was 42.28 ± 6.14 Pa, and that of seven-day spheroids was in the range 62.40 ± 5.58 Pa^61^. However, these approaches cannot provide high-speed measurements of the mechanical properties of a large number of spheroids.

The described method of nanoindentation significantly accelerates the process of obtaining relevant data on spheroids’ material properties, due to the high speed and ease of analysis over the AFM. This method uses local (small-scale) non-destructive indentation, provides the possibility of working in liquid media at 37 °C and does not require extensive sample preparation prior to testing, the sample needs only to be immobilized. The proposed technique of spheroid fixation at the plate surface makes them immobile and allows the surface layers of spheroids to be studied. Furthermore, nanoindentation allows for the measurement of minimal forces and displacements, generally in the range of μN–mN and nm–μm, respectively, and if necessary provides fast and not-noisy dynamic mechanical analysis to obtain data on viscoelastic properties.

Under the conditions of the 2D monolayer, cells are located on a rigid surface of culture plastic, which stimulates the migratory phenotype associated with planar polarity, reconstruction of the cytoskeleton and intercellular junctions, loss of apical-basal polarity and epithelial-mesenchymal transition^62^. First, we studied cells isolated from human eye tissues, mesenchymal L-MSCs and epithelial RPE cells, in a 2D culture. The properties of L-MSC cells were in agreement with those reported previously^16,63^. The RPE tissue was discovered more than a century ago, and in vivo it displays the anatomical features of a typical monolayer epithelium with tightly packed cuboidal-to-columnar cells and apical microvilli^64^. A challenge that still remains is to determine how this characteristic RPE phenotype changes in culture, and there are still no standard criteria for describing the RPE cell phenotype in vitro^14^. While the classical epithelial phenotype with ZO-1 + tight junctions is preserved in the RPE cell cultures (Fig. 1D,E), at the fourth passage they also have mesenchymal markers: CD29 in 99.9% of cells, CD105 in 58.9% and CD90 in 83.1%. The hematopoietic marker CD45 was expressed in 43.5% of cells, and CD19 (the marker of B lymphocytes and follicular dendritic cells^65^) was present in 40% of RPE cells (Table 1, Supplementary Fig. S2), which is in agreement with previous reports showing that hematopoietic cell markers are constitutively expressed in RPE cells and may influence the macrophage-like properties of these cells^66^.

In a 2D monolayer, about half of the cell membrane area interacts with the culture plate surface, another half faces toward the cell medium, and only a small percentage of membrane area contains intercellular junctions^67^. As a result, cells cannot reproduce the native positioning of intercellular junctions, loss of apical-basal polarity takes place, the cytoskeleton structure is modified^68^, and proliferative activity is higher in these cells. Since cell shape is an important factor that regulates growth, physiology, development, and differentiation, mostly due to a reorganization of the actin cytoskeleton, 3D cell culture is becoming more and more widespread. Growing cells in a 3D environment leads to the formation of more intercellular junctions, which stops active proliferation, protects cells from apoptosis^69^ and promotes activation of functional genes^70–72^. In spheroids, the cells gain resistance to hypoxia and some external stresses and actively synthesize extracellular matrix (ECM)^62^. For L-MSCs and RPE cells, the formation of compact spheroids took seven days, which is in agreement with the previously described techniques for the establishment of standard, easily scalable self-organizing 3D spheroids from human somatic cells^73,74^.

Formation of spheroids is a complex process that depends on initial cell phenotype^75,76^. In spheroids from L-MSCs, two zones could be distinguished: the outer zone consisting of four to five outer flattened cell layers with a well-developed cytoskeleton and tiled organization, and the inner area comprising sparsely located polygonal cells with a large quantity of ECM in between (Fig. 2A–C, Supplementary Fig. S3A, B). Cells in L-MSC spheroids expressed the mesenchymal marker vimentin, and the accumulation of ECM components such as laminin was observed (Fig. 3B). Such ECM accumulation in spheroids from limbal cells was also noted in previous studies^77^. Expression of CD11b integrin increased to 25.7% and CD19 to 15.7% in spheroids (Supplementary Fig. S7). The presence of these surface antigens has been shown before for several MSCs from various sources, and they may help cells to respond efficiently to transmembrane signals^78^. It is possible that ECM accumulation, high expression of the adhesion molecules and increased synthesis of the CD19 signal molecule are the key factors in the reconstruction of the complex and stable niche that supports the structural and functional potential of L-MSCs and effective overall spheroid organization.

The formation of intercellular junctions in 3D cultures is one of the main processes that determine cell behavior, allows recovery and sustainability for the epithelial cell phenotype and controls the tissue structural organization^59^. Tight junctions (ZO-1+, Fig. 3C) were formed between the surface cells in RPE spheroids, their apical-basal polarity was partially recovered, and microvilli were present on the apical surface. The cells of the inner zone were distributed more sparsely, while ECM was almost absent in this zone (Fig. 2D–E, Supplementary Fig. S3C). Expression of adhesion molecules was elevated in RPE spheroids, compared to the 2D culture: 20.8% of cells expressed CD11b integrin, 98.5% expressed CD90, and 74% expressed CD105 (Table 1, Supplementary Fig. S8). The expression of CD34 in 11.4% of cells could be the result of cell adaptation to 3D culture conditions^57^.

## Conclusion

In summary, we have experimentally shown that the existing models of cell self-assembly do not entirely capture the spheroid fusion process. We believe this is due to such biophysical processes as ECM remodeling and different cell migration modes. We applied the nanoindentation technique for analysis of the mechanical properties of spheroids obtained from cells with different phenotypes. The findings on the mechanical stiffness of spheroids may provide new data to support their eventual application. Further studies of spheroid fusion mechanisms will help to optimize the process of obtaining complex cell-based constructs consisting of different types of cells, which will serve a convenient tool in the field of tissue engineering.

## Materials and methods

### 2D and 3D cell cultures

The study was conducted on two primary human somatic cell cultures of mesenchymal (limbal mesenchymal stem cells—L-MSCs) and epithelial (retinal pigment epithelium—RPE cells) phenotypes. Biopsies of the post-mortem eye tissues were kindly provided by the cryobank of the Center for Fundamental and Applied Biomedical Problems of the S. Fyodorov Eye Microsurgery Federal State Institution. All the procedures were approved by the Ethical Committee of Federal State Budgetary Scientific Institution ‘Institute of General Pathology and Pathophysiology’, while performed in accordance with the Helsinki Declaration.

L-MSCs were isolated from corneoscleral rings—limbal stroma of the eye. RPE cells were obtained from retinal pigment epithelium samples. Primary cultures of L-MSCs and RPE cells were obtained by previously described techniques^76,79^. In brief, tissue samples were washed thrice in sterile Hanks’ solution (Biolot, St.-Petersburg) supplemented with antibiotics (gentamicin, penicillin–streptomycin) for 15–20 min. Explants were then placed in Hanks’ solution, mechanically cut into small pieces and placed in Petri dishes (35 mm, SPL) in a small volume of a medium under standard culture conditions (37 °C, 5% CO_2_), to provide the adhesion of explants to tissue culture plastic.

The culture medium for L-MSCs consisted of DMEM/F12 (1:1, Biolot, St.-Petersburg) supplemented with l-glutamine (2 mM, Paneco, Moscow), gentamicin (50 µg/ml, Paneco, Moscow), insulin-transferrin-selenium (1:100, Biolot, St.-Petersburg), 20 ng/ml bFGF (ProSpec, Israel) and 10% fetal bovine serum (FBS, HyClone, USA). RPE cells were cultured in DMEM/F12 (1:1), supplemented with l-glutamine (2 mM, Paneco, Moscow), gentamicin (50 µg/ml, Paneco, Moscow), 20 ng/ml bFGF (ProSpec, Israel), 20 ng/ml EGF (ProSpec, Israel), 1% N2/B27 supplement (Gibco, USA) and 5% FBS (HyClone, USA).

Cell cultures’ morphology and confluency were assessed visually every two to three days under an inverted phase-contrast microscope CKX41 (Olympus, Japan). The cell culture medium was replaced two or three times per week. When the cell monolayer reached 100% confluency, cultures were passaged using versene and 0.25% trypsin solutions. Trypsin activity was then blocked by growth medium; cell concentration during passaging was more than 10^5^ cells/ml.

The characterized 2D cultures were used to produce 3D cultures—cell spheroids, using the previously developed standard culture technique on agarose plates 75. Cell suspensions were obtained using versene and 0.25% trypsin solutions and centrifuged (7 min, 400 g). The pellets were resuspended in growth medium up to a concentration of 3 × 10^6^ cells/ml and then placed on non-adhesive agarose plates formed using a 3D Petri Dish (Microtissues, USA). Agarose plates were transferred into 12-well culture plates (SPL, Korea) with the growth medium required for the cell type and cultured under standard conditions (37 °C, 5% CO_2_) for seven days.

### Cell spheroid fusion

L-MSCs and RPE-cell spheroids were co-cultured in a “hanging drop” system. Here, 30-µL drops of complete growth medium were placed on the non-adhesive Petri dish cover, while the dish itself was filled with the same medium, to prevent drop evaporation and retain the osmolarity. The pairs of seven-day spheroids, L-MSCs + L-MSCs, RPE + RPE cells and L-MSCs + RPE cells, were placed in the drops under the control of a stereomicroscope (Olympus SZX16, Japan) and incubated under standard conditions (37 °C, 5% CO_2_). For the L-MSCs + RPE-cell spheroid pair, a mixed growth medium was used. The images were taken after 0.5, 1, 2, 3, 4, 6, 7, 24 and 48 h of incubation, using the digital camera DP 12-2 (Olympus, Japan) of the stereomicroscope. The initial spheroid radii and the neck radius were measured with ImageJ software (NIH, Bethesda, MD). 12 fusion pairs were analyzed for each case.

### Flow cytometry and immunocytochemistry

The immunophenotype of the cell cultures was studied using techniques of flow cytometry and immunocytochemical staining. Cells after trypsinization and centrifugation (7 min, 400 g) were resuspended in PBS (pH 7.4) supplemented with 1% FBS, to obtain 700-μl aliquots to analyze the expression of surface markers (CD11b, CD14, CD19, CD29, CD34, CD90, CD45, and CD105). Antibodies conjugated with fluorescent labels (FITC—fluorescein isothiocyanate, PE—phycoerythrin and APC—allophycocyanin) were added to samples (10 µl of antibody per 1 × 10^6^ cells) and incubated in the dark (15 min, 25 °C). Samples were then centrifuged again (5 min, 400 g) and the pellets were resuspended in 1 ml of PBS containing 1% FBS. The analysis was performed on a Cytomics FC 500 flow cytometer (Beckman Coulter, Inc., USA). Each measured sample consisted of 1 × 10^6^ cells for monolayer culture and 750 spheroids for 3D culture; 10,000 events were analyzed in each case.

For immunocytochemical staining, monolayer cultures were seeded on coverslips and spheroids were fixed in 4% paraformaldehyde solution (20 min, 4ºC), washed thrice from fixator in PBS (pH 7.4) and incubated overnight at + 4 °C with primary antibodies against ZO-1, vimentin, nestin, laminin and fibronectin (Thermo Scientific, USA). Primary antibody solutions contained 0.15% Triton X-100 to improve membrane permeability. Samples were then washed thrice in PBS (pH 7.4) and incubated with secondary antibodies conjugated with Fluorescein, Alexa Fluor 488 and Alexa Fluor 594 (Thermo Scientific, USA) at room temperature in the dark for 1 h. Cell nuclei were counterstained with bisbenzimide (Hoechst 33258, Serva). Stained samples were mounted in VitroGel medium (BioVitrum, Moscow) and analyzed under a laser scanning confocal microscope Olympus FLUOVIEW FV10i (Olympus, Japan).

### Histological analysis and electron microscopy

To analyze the structure and morphology of spheroids, the samples were fixed in glutaraldehyde (3% solution in PBS, pH 7.4) for 1 h at room temperature or overnight at + 4 °C. The samples were washed from the fixative thrice in PBS and fixed in OsO_4_ (1% solution in PBS, pH 7.4) for 1 h at room temperature. The samples were then washed thrice again in PBS and dehydrated in alcohols of ascending concentrations, at 50°, 70° and 80° (twice for 5 min each), 96° (twice for 20 min), 100° (20 min) and acetone (twice for 5 min). For further sample processing, an epoxy resin consisting of EMbed-812, Araldite 502, DDSA and DMP30 catalyst (Electron Microscopy Sciences, USA) was prepared. For 100 ml of resin, 25 ml of Araldite GY, 15 ml of Araldite 502, 55 ml of DDSA and 1.6 ml of DMP30 were used. The obtained resin was pre-incubated for 1 h at 37 °C. Samples after the second shift of acetone were placed in a mixture of acetone and resin (1:1) for 1 h at room temperature in closed Eppendorf tubes, then transferred to embedding capsules for 1 h at room temperature, to form blocks of pure resin. The samples were then incubated at + 60 °C for three to five days to provide full resin polymerization. Semi-fine sections (1 µm thick) were obtained on a Leica EM UC6 ultramicrotome (Leica, Austria), stained for 10 min with toluidine blue and sodium tetraborate and differentiated under running water. The resulting sections were analyzed under a bright-field Olympus BX51 microscope (Olympus, Japan) equipped with a ColorView II camera. Cell F software was used for photo registration.

For transmission electron microscopy, ultrathin sections were made from the blocks of spheroids embedded in pure resin, using an LKB-111 ultramicrotome (Sweden), and contrasted in 1% uranyl acetate solution in distilled water for 1 h at room temperature in the dark and lead citrate, according to the previously described protocol^80^. The samples were studied using a JEM-1011 TEM (JEOL Ltd, Japan).

To study the samples using scanning electron microscopy, fixed and dehydrated spheroids were exposed to critical-point drying, mounted on an object table and overlaid with fine gold particles. The resulting replica was analyzed under a Camscan S2 scanning electron microscope (Cambridge Instruments, England).

### Mechanical analysis

Nanoindentation was performed on monolayer cultures at the third to fourth passages and seven-day spheroids formed from the same cultures. The mechanical properties of samples were measured using a Piuma Nanoindenter (Optics11, Netherlands). Indenter probes were used with a cantilever stiffness ranging from 0.041 to 0.05 N/m, together with a spherical tip with a radius of 9 μm. During every indentation, the probe tip was dipped into the sample to a depth of 3.5 μm, followed by 1 s of dwell time (the piezo position was held constant) and retraction. All indentations were performed in 35-mm Petri culture dishes (SPL, Korea) placed on a thermostatic plate at 37 °C. During indentation, all the samples were kept in the full growth media required for each cell type supplemented with 15 μl/ml HEPES to maintain a pH of 7.4. The duration of measurement of one sample series did not exceed 40 min, to provide cell viability. Monolayer cell cultures (L-MSCs and RPE cells) at the fourth passages were analyzed in automatic mode. For each sample of the cell monolayer, we measured three areas of 250 × 250 μm with 25-μm steps along the axes.

Petri dishes covered with poly-l-lysine (Sigma) according to the manufacturer’s protocol were used for cell spheroids fixation on the surface during the measurements. Spheroids in a small volume of growth medium were placed in prepared Petri dishes to improve their adhesion to the surface. After 2 or 3 min, the growth medium was added in the amount required for the cantilever immersion. Due to the spherical shape of the spheroids, the measurements of Young’s modulus were performed in the central area of their surface, where the direction of indentation was perpendicular to the surface of the spheroid (Supplementary Fig. S9). Indentations were carried out for twenty spheroids in each of two series of different cell-type spheroids; each spheroid was indented three or four times.

The load–displacement curves obtained during the indentation were processed using Piuma Dataviewer software. The loading part of the curve from the point where the tip touches the surface (contact point) to a depth equal to 10% of the spherical tip radius (900 nm), was processed using Hertz’s model to obtain the Young’s modulus *E*:

4 $$ F = \frac{4}{3}\frac{E}{{1 - \nu^{2} }}\delta^{3/2} \sqrt R , $$

where *F* is the measured force, and *δ* is the indentation depth. A radius of the spheroids (50 μm) was substantially larger than a radius of the used spherical probe (*R* = 9 μm), and the indentation depth was much smaller than the spheroid size (< 5%). Thus, the model considers the spheroid as a half-space and assumes that deformation at the interface of the spheroid and the bottom surface is negligibly small^81^. The Poisson’s ratio *ν* was set to 0.5. The same considerations were applied for the cell monolayer indentation. Load-indentation curves were processed only up to 900 nm depth, which is much smaller than the thickness of cells in 2D culture (4–6 μm), and allowed us to diminish the impact of the stiff substrate on the obtained data (Supplementary Fig. S10).

Additionally, the complete indentation curve (loading, dwell and retraction parts) was processed in MATLAB software via a previously described numerical algorithm^44^, to extract viscoelastic properties. Briefly, the algorithm utilizes Ting’s solution for the problem of the contact of the sphere with a viscoelastic material that can be characterized by a relaxation function (Young’s relaxation modulus). The relaxation function for the power-law rheology (PLR) model was selected in this study (Eq. 1).

### Statistical analysis

Statistical analyses were performed using Statistica 10.0 software (StatSoft, USA). Data were subjected to the Shapiro–Wilk χ^2^ and Pearson normality tests. A one-way analysis of variance (ANOVA with Bonferroni’s multiple comparison) test was employed to determine statistical differences between the means of several independent groups. The data are presented as mean ± standard deviation, where applicable.

## Supplementary information

Supplementary information 1.

Supplementary information 2.
