# Author Correction: Cell spheroid fusion: beyond liquid drops model

**DOI:** 10.1038/s41598-021-94254-w

**Published:** 2021-08-02

**Authors:** Nastasia V. Kosheleva, Yuri M. Efremov, Boris S. Shavkuta, Irina M. Zurina, Deying Zhang, Yuanyuan Zhang, Nikita V. Minaev, Anastasiya A. Gorkun, Shicheng Wei, Anastasia I. Shpichka, Irina N. Saburina, Peter S. Timashev

**Affiliations:** 1 FSBSI “Institute of General Pathology and Pathophysiology”, 8, Baltiyskaya st., Moscow, 125315 Russia; 2 FSBEI FPE “Russian Medical Academy of Continuous Professional Education” of the Ministry of Healthcare of Russia, 2/1, Barrikadnaya St., Moscow, 125993 Russia; 3 Faculty of Biology, Lomonosov Moscow State University, 12-1, Leninskie Gory, Moscow, 119234 Russia; 4 Institute for Regenerative Medicine, Sechenov First Moscow State Medical University, 8-2, Trubetskaya St., Moscow, 119991 Russia; 5 Institute of Photonic Technologies, Research Center “Crystallography and Photonics” RAS, 2, Pionerskaya st., Troitsk, Moscow, 142190 Russia; 6 Department of Urology, Children’s Hospital of Chongqing Medical University, Chongqing, People’s Republic of China; 7 Wake Forest University Institute for Regenerative Medicine, Winston-Salem, NC USA; 8 Department of Oral and Maxillofacial Surgery/Central Laboratory, Peking University School and Hospital of Stomatology, Beijing, 100081 People’s Republic of China; 9 Laboratory of Biomaterials and Regenerative Medicine, Academy for Advanced Interdisciplinary Studies, Peking University, Beijing, 100871 China; 10 Department of Polymers and Composites, N.N. Semenov Institute of Chemical Physics, 4, Kosygin st., Moscow, 119991 Russia; 11 Chemistry Department, Lomonosov Moscow State University, 1-3, Leninskiye Gory, Moscow, 119991 Russia

Correction to: *Scientific Reports* 10.1038/s41598-020-69540-8, published online 28 July 2020

The original version of this Article contained an error in the spelling of the author Anastasia I. Shpichka which was incorrectly given as Anastasia A. Shpichka.

The original Article and accompanying Supplementary Information file have been corrected.

