# Supplementary material for: Cell spheroid fusion: beyond liquid drops model
Source: Sci Rep. 2020 Jul 28;10:12614. doi: 10.1038/s41598-020-69540-8 (PMC7387529; doi:10.1038/s41598-020-69540-8)
Supplement: Supplementary file 1 — Supplementary information 1. [file 41598_2020_69540_MOESM1_ESM.pdf]

Cell Spheroid Fusion: Beyond Liquid Drops Model

Nastasia V. Kosheleva[1,2,3], Yuri M. Efremov[4], Boris S. Shavkuta[4,5], Irina M. Zurina[1,2,4], Deying Zhang[6], Yuanyuan Zhang[7], Nikita V. Minaev[5], Anastasiya A. Gorkun[1,2,4], Shicheng Wei[8,9], Anastasia I. Shpichka[4], Irina N. Saburina[1,2] and Peter S. Timashev[4,5,10,11]

[1] FSBSI "Institute of General Pathology and Pathophysiology", 8, Baltiyskaya st., Moscow, 125315, Russia
[2] FSBEI FPE "Russian Medical Academy of Continuous Professional Education" of the Ministry of Healthcare of Russia, 2/1, Barrikadnaya st., Moscow, 125993, Russia
[3] Lomonosov Moscow State University, Faculty of Biology, 12-1, Leninskie Gory, Moscow, 119234, Russia
[4] Sechenov First Moscow State Medical University, Institute for Regenerative Medicine, 8-2, Trubetskaya st., Moscow, 119991, Russia
[5] Institute of Photonic Technologies, Research center "Crystallography and Photonics" RAS, 2, Pionerskaya st., Troitsk, Moscow, 142190, Russia
[6] Department of Urology, Children's Hospital of Chongqing Medical University, Chongqing, People's Republic of China
[7] Wake Forest University Institute for Regenerative Medicine, Winston-Salem, NC, United States of America
[8] Department of Oral and Maxillofacial Surgery/Central Laboratory, Peking University School and Hospital of Stomatology, Beijing, 100081, China
[9] Laboratory of Biomaterials and Regenerative Medicine, Academy for Advanced Interdisciplinary Studies, Peking University, Beijing, 100871, China.
[10] Department of Polymers and Composites, N.N. Semenov Institute of Chemical Physics, 4, Kosygin st., Moscow, 119991, Russia
[11] Lomonosov Moscow State University, Chemistry Department, 1-3, Leninskiye Gory, Moscow 119991, Russia

E-mail: n_kosheleva@mail.ru

Supplementary Information

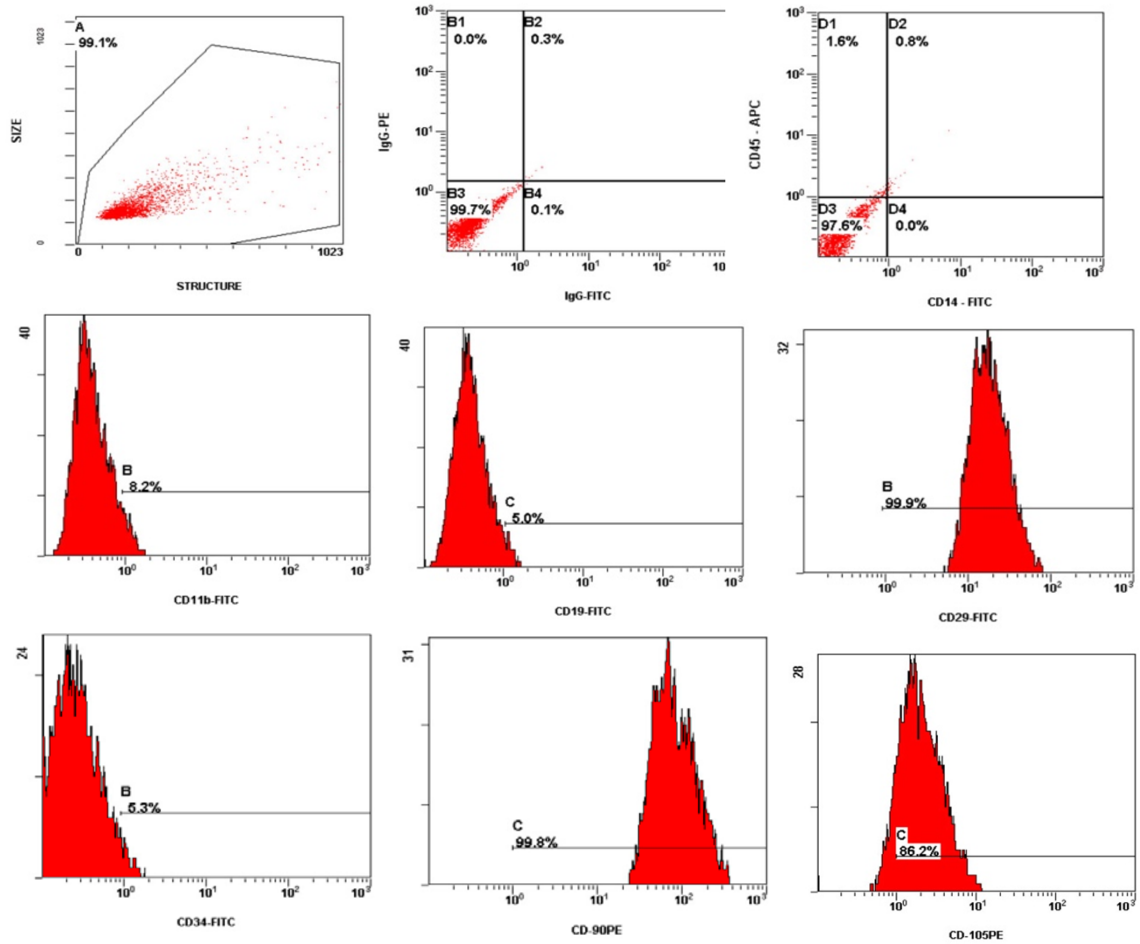

FigureS1. Immunophenotypic profile of 2D L-MSC culture at passage 4.

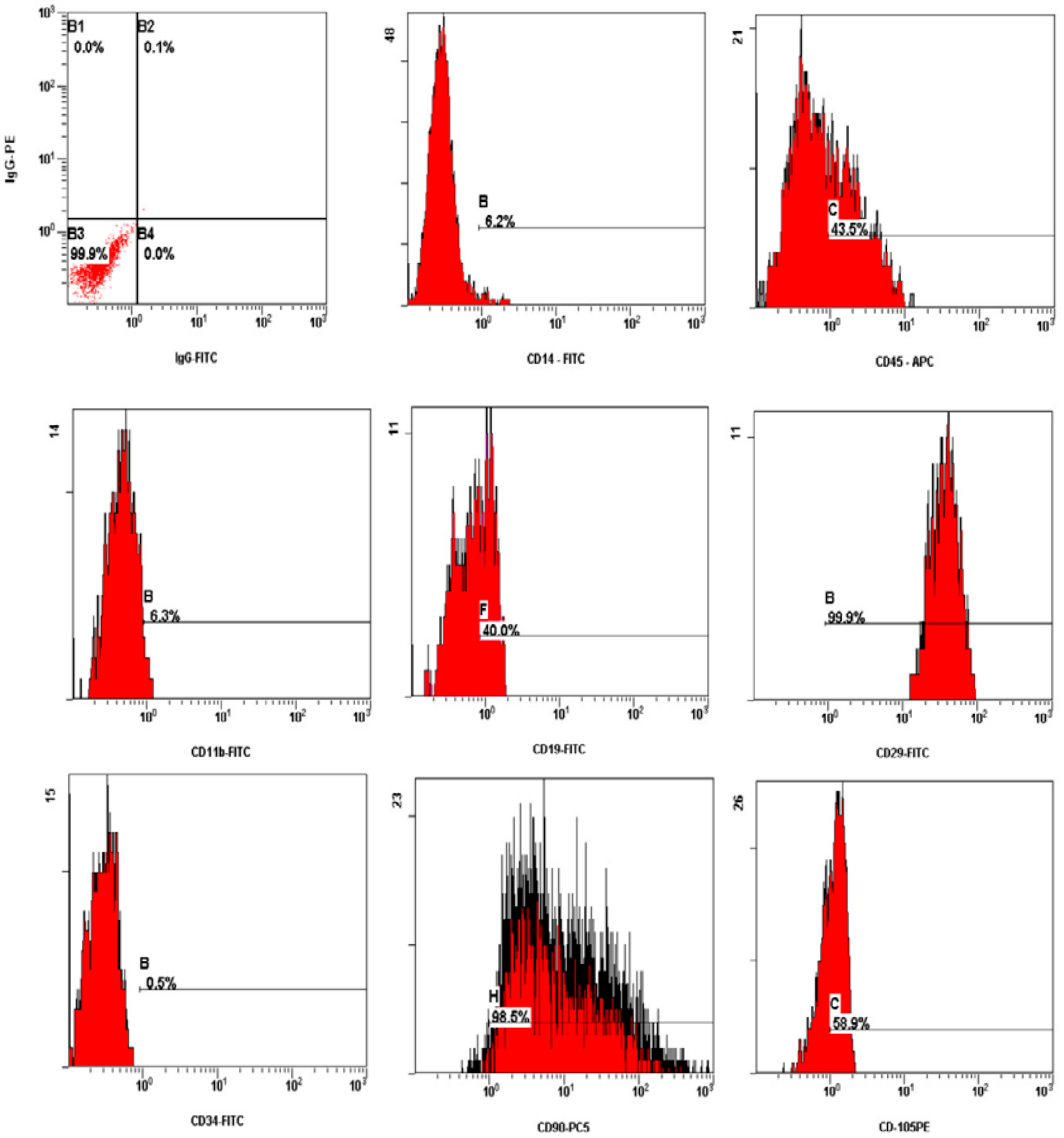

Figure S2. Immunophenotypic profile of 2D RPE culture at passage 4.

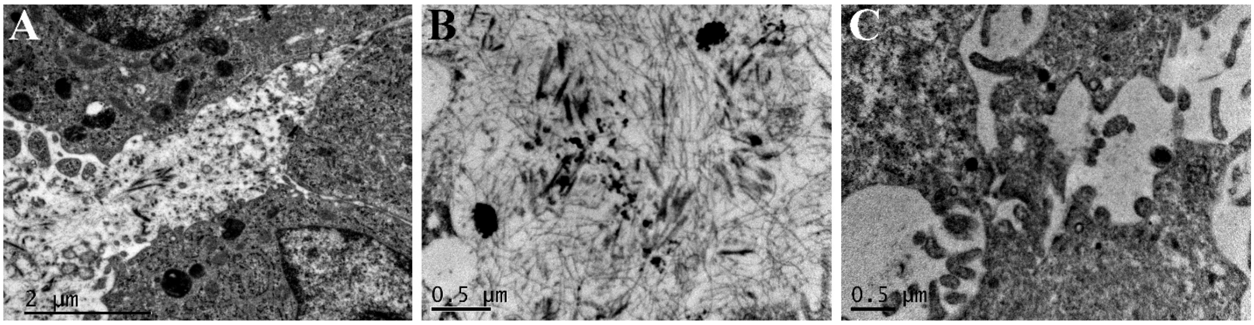

Figure S3. Structure of inner zone of seven-day-old spheroids from the L-MSCs (A, B) and RPE cells (C). (A) – inner zone cells of L-MSC spheroids were round or polygonal, embedded in the extracellular matrix; (B) – extracellular matrix in the inner zone of the L-MSC spheroids; (C) – in the inner zone of RPE cells, spheroids with polygonal or round cells were distributed loosely with a low amount of extracellular matrix. *Transmission electron microscopy.*

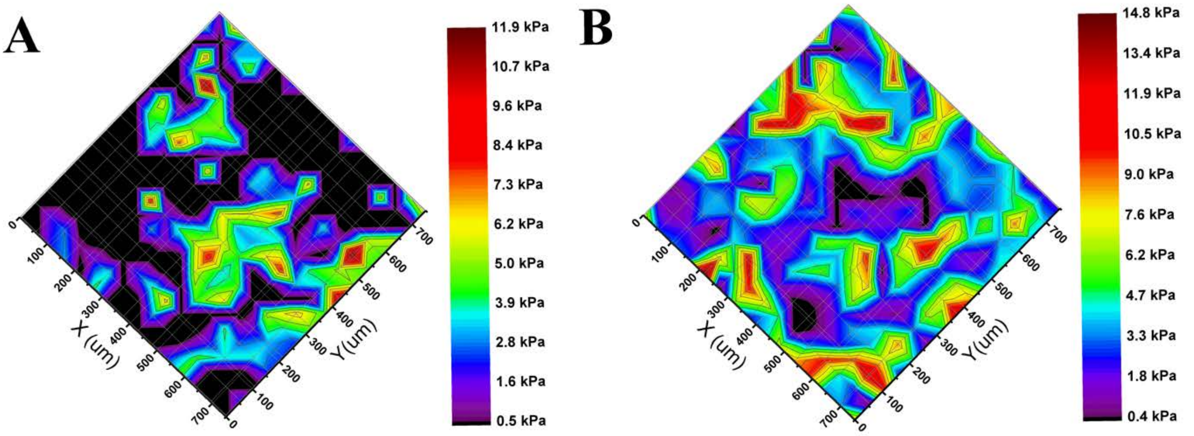

Figure S4. Distribution of Young's modulus in L-MSCs (A) and RPE cells (B) monolayer cultures. Black color refers to the surface of the tissue culture plastic.

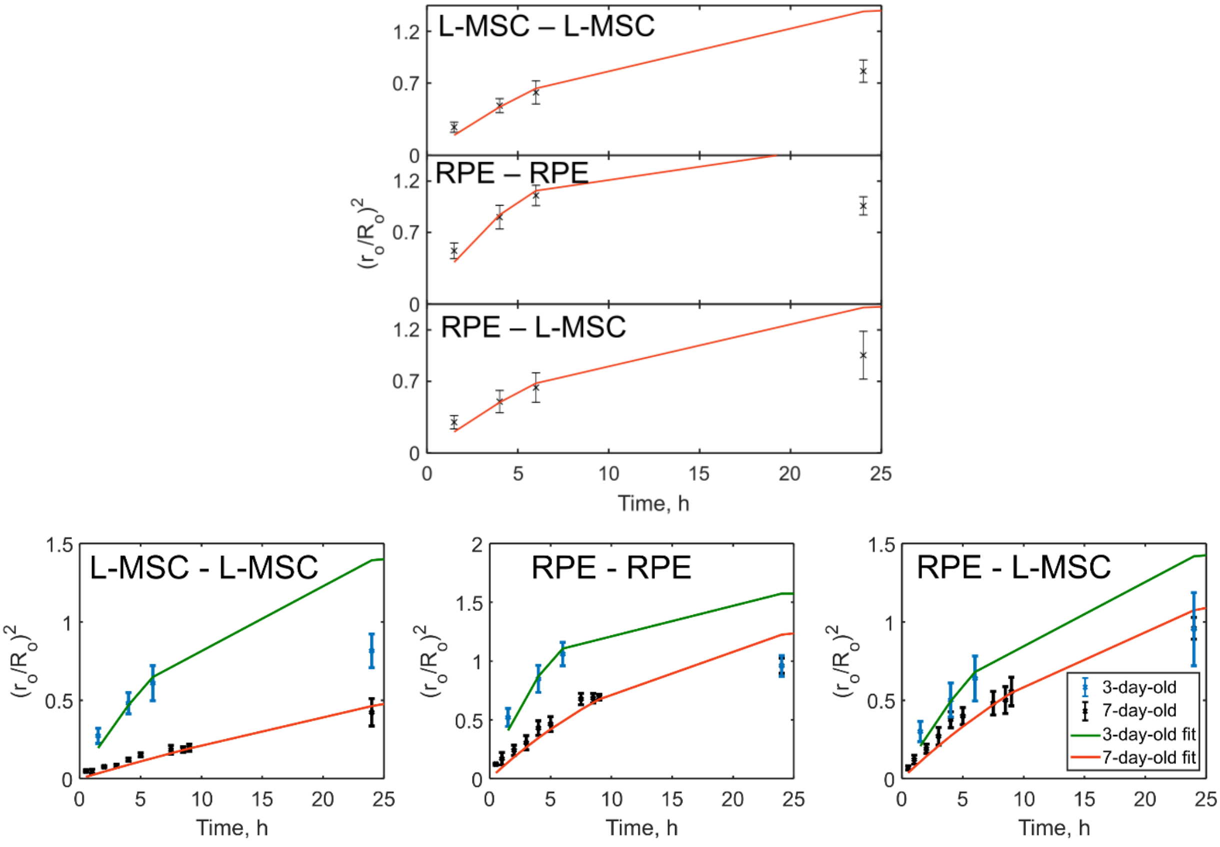

Figure S5. Fusion of the 3-day-old spheroids analyzed with the model for liquid drops. Time evolution of the parameter $(r_0/R_0)^2$ during the fusion of two L-MSCs cells (top), two RPE (middle) and an RPE-L-MSC pair of spheroids; an average value of 5 different spheroid pairs (vertical bars represent SDs). Fitting of the data sets with the model is shown with red curves, only first three data points were used for fitting (up to 6 h). (The constants are 12±5, 7±1, 9±2 h, Adj. R^2 values are 0.96, 0.91, 0.96 for L-MSC - L-MSC, RPE - RPE and RPE - L-MSC, respectively). Bottom row: comparison of the fusion of the 3-day-old and 7-day-old spheroids. 3-day-old spheroids demonstrate much faster fusion (the curve lies higher), especially for the L-MSC - L-MSC pair.

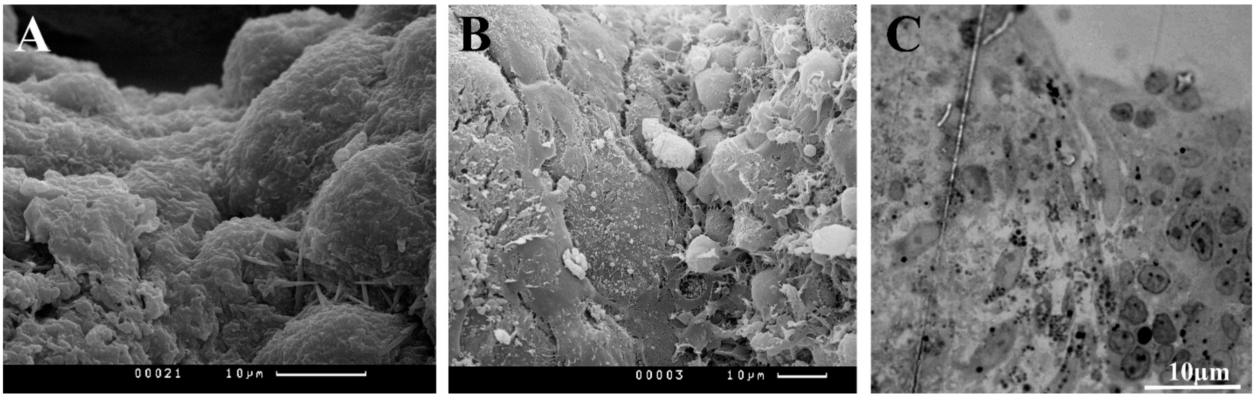

Figure S6. Structure of neck zone of fusing spheroids – two L-MSC spheroids, 24 h after fusion (A), and L-MSC with RPE-cell spheroids, 3 h after fusion (B, C). (A) – slow remodeling of L-MSCs shape in the process of fusion; (B, C) – fast collective migration of RPE cells from epithelial spheroid (right side) on the surface of the flattened outer zone of L-MSC spheroid (left side).

A, B - *scanning electron microscopy*; C – *semi-fine section, light microscopy*.

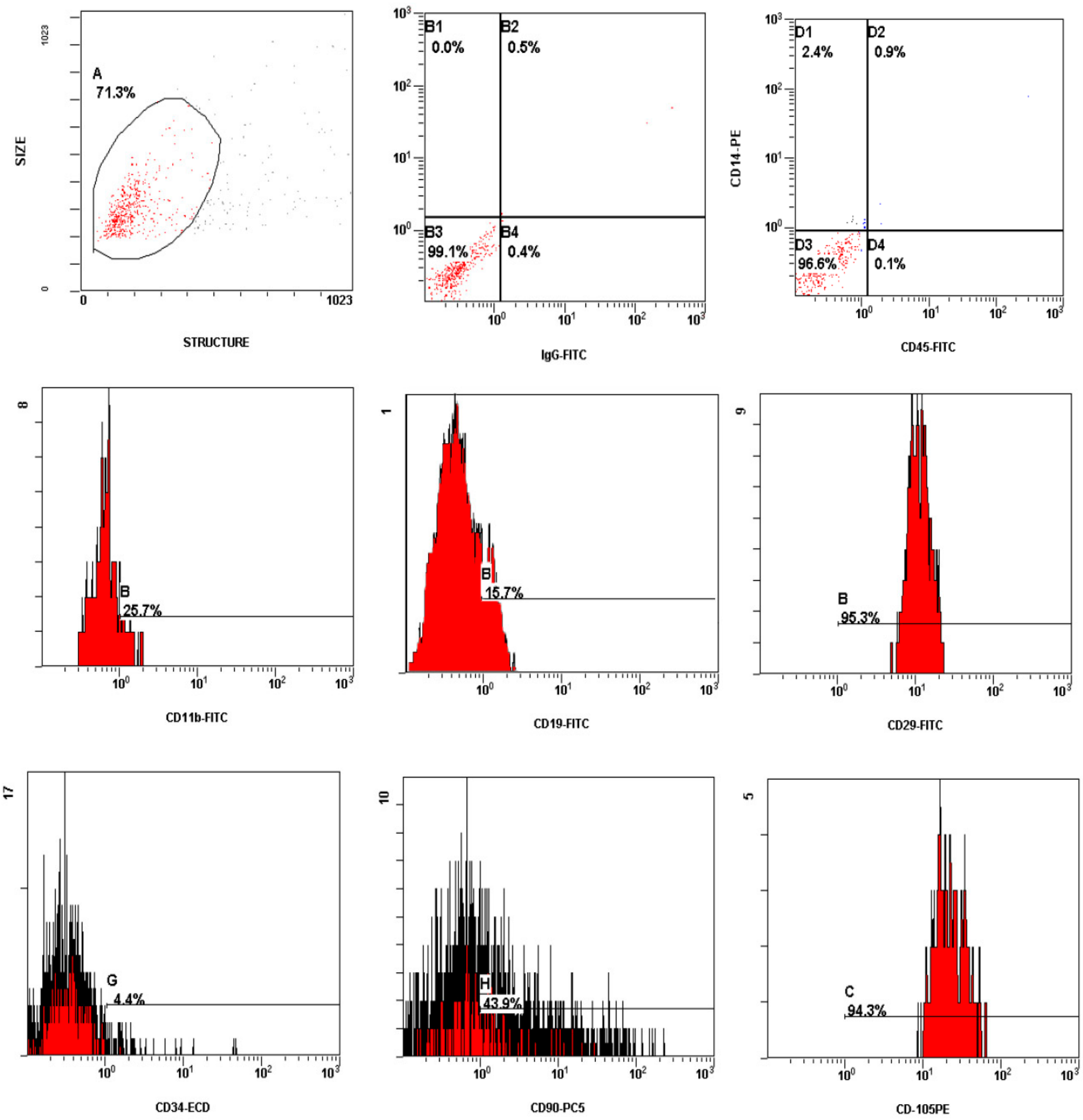

Figure S7. Immunophenotypic profile of 3D L-MSC culture

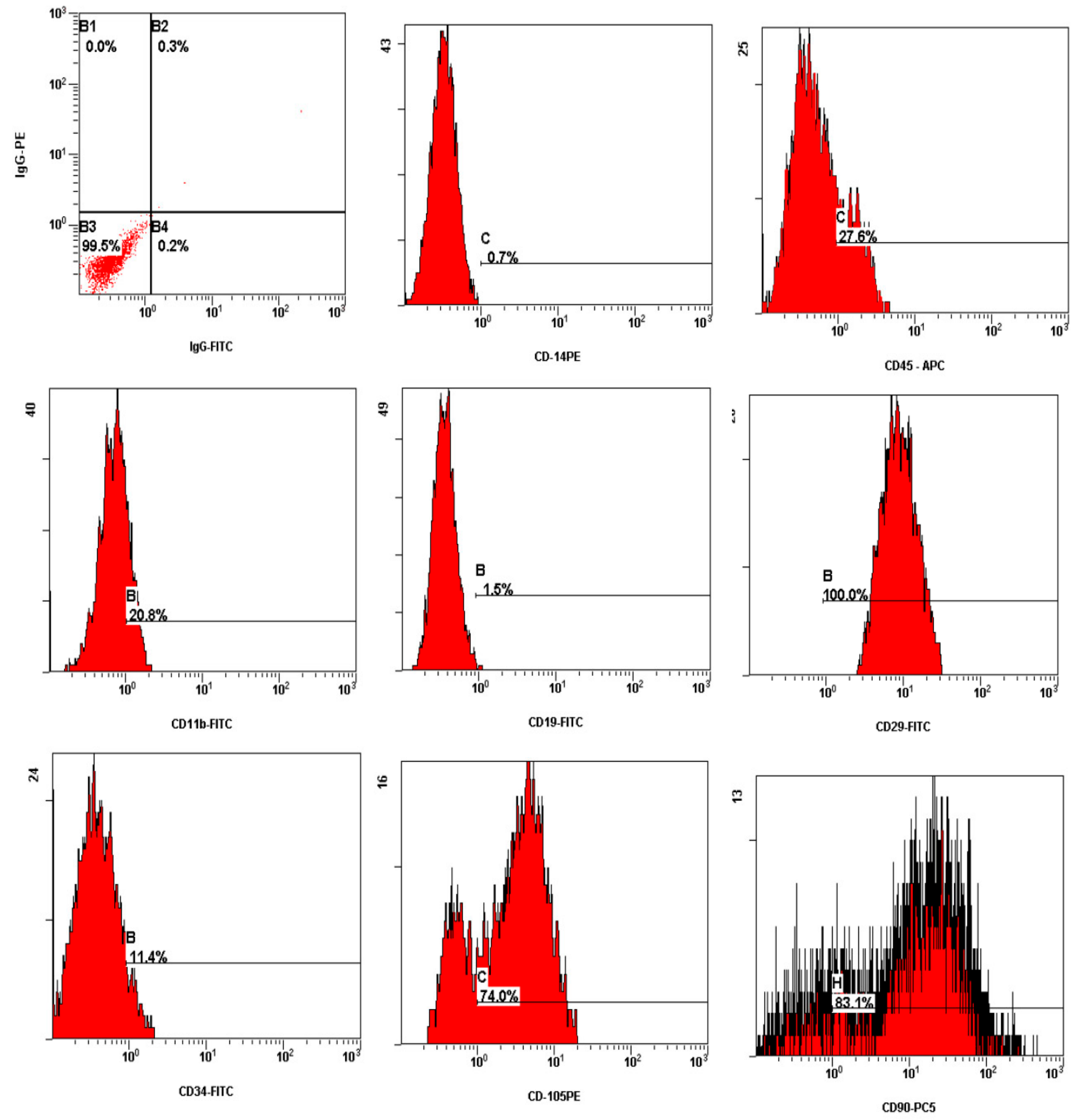

Figure S8. Immunophenotypic profile of 3D RPE culture

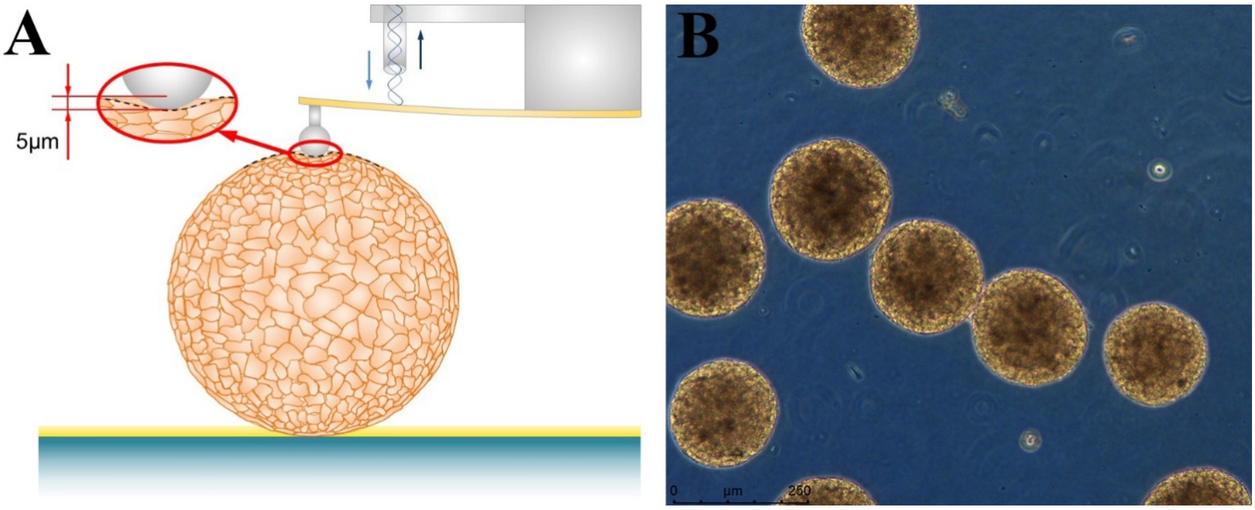

Figure S9. Nanoindentation of cell spheroids. (A) – scheme of the indentation experiments. Spheroid is attached to the polylysine-modified surface (yellow) of the tissue culture plastic (blue). The spherical probe at the end of the cantilever indents the surface to a depth of 3-5 μm and the cantilever deflection is measured by the interferometer. (B) – RPE-cell spheroids on the surface prepared for the indentation experiment. *Light phase-contrast microscopy*.

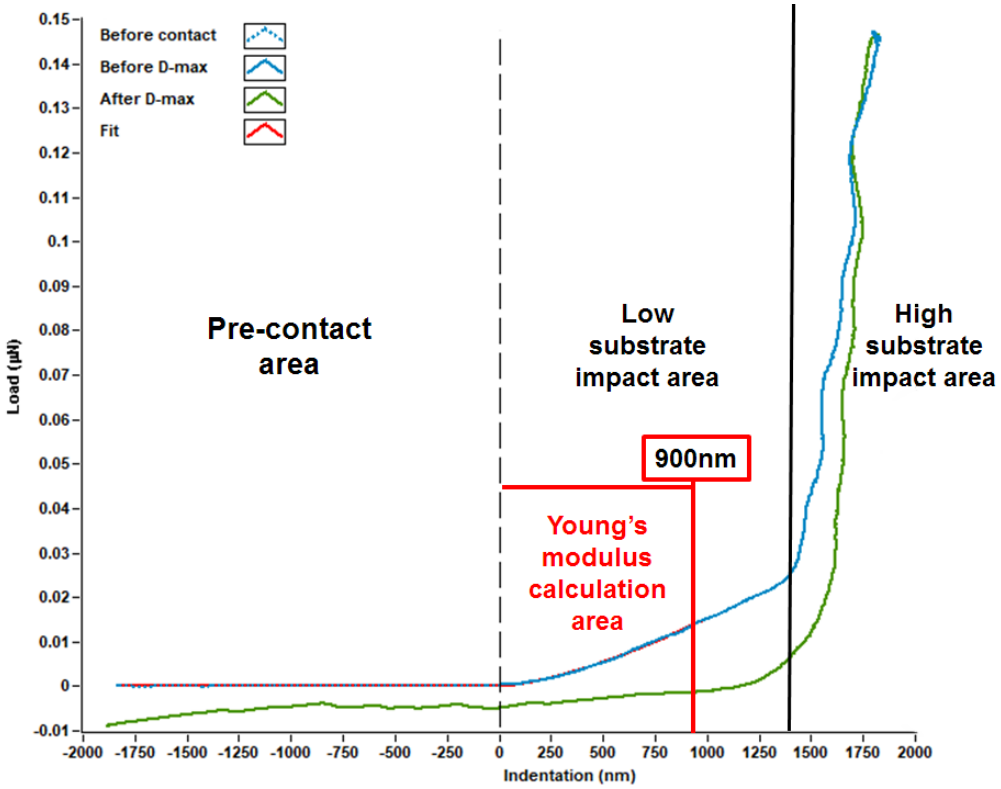

Figure S10. Typical load-indentation curve received during cell monolayer nanoindentation. Young's modulus calculations proceed only in the red area (indentation depth of it is lower than 900nm, that is 10% of tip radius), where substrate does not affect the results.

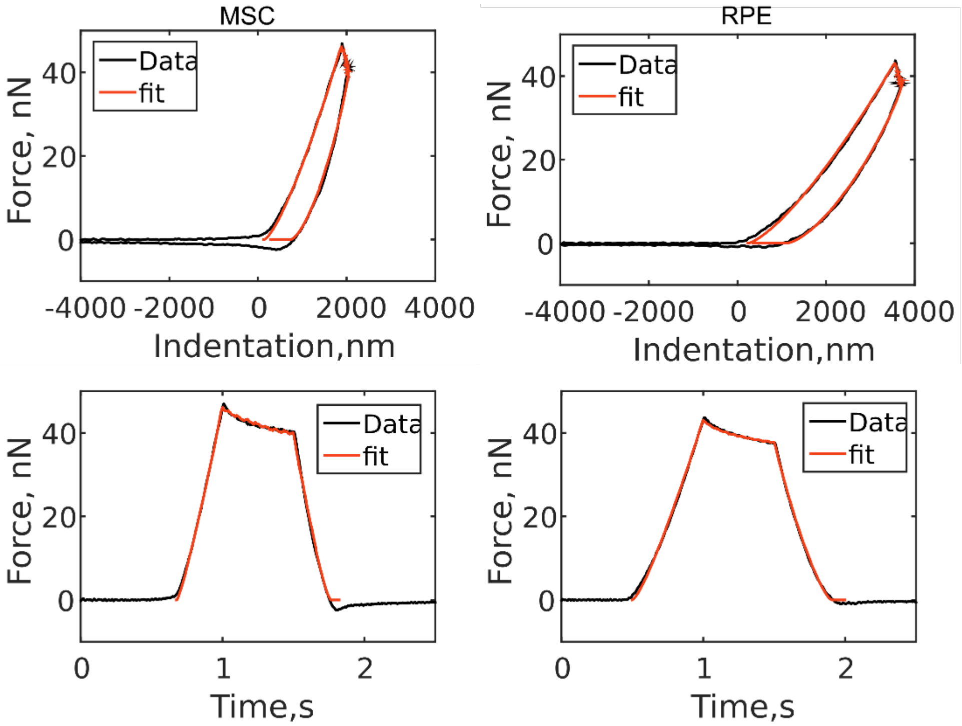

Figure S11. The experimental force-distance (force-time) curves obtained for L-MSC and RPE seven-day-old spheroids. Fitting of the complete indentation region with the viscoelastic power-law rheology model is shown.
